# F-18 FDG PET, CT, and MRI for detecting the malignant potential in patients with gastrointestinal stromal tumors: A protocol for a network meta-analysis of diagnostic test accuracy

**DOI:** 10.1097/MD.0000000000010389

**Published:** 2018-04-20

**Authors:** Kongyuan Wei, Bei Pan, Huan Yang, Cuncun Lu, Long Ge, Nong Cao

**Affiliations:** a Department of General Surgery, First Affiliated Hospital of Lanzhou University; b Department of Social Medicine and Health Management, School of Public Health; c Evidence-based Medicine Center, School of Basic Medical Sciences, Lanzhou University, Lanzhou, China.

**Keywords:** CT, F-18 FDG PET, gastrointestinal stromal tumors, MRI, network meta-analysis, sensitivity, specificity

## Abstract

**Background:**

Gastrointestinal stromal tumor (GIST) is a rare cancer in gastrointestinal carcinomas and has been widely known as a curable disease among all the digestive tumors. However, early detection of malignant potential in patients with GIST has still been a huge challenge all around the world. CT, MRI, and F-18 FDG PET are all considered as good tests for diagnosing malignant GIST efficiently, but no recommended suggestions presents which test among the 3 is the prior one in detecting the malignant potential of GIST. We perform this study to assess the accuracy between CT, MRI, and F-18 FDG PET through network meta-analysis method, and to rank these tests.

**Methods and analysis:**

PubMed, EMBASE.com, CNKI, and CBM databases will be searched without search date and language restrictions. We will include diagnostic tests which assessed the accuracy of CT, MRI, and F-18 FDG PET in detecting the malignant potential of GIST. The risk of bias in each study will be independently assessed as low, moderate, or high using criteria adapted from Quality Assessment of Diagnostic Accuracy Studies 2 (QUADAS-2). Meta-analysis will be performed using STATA 12.0 and R 3.4.1 software. The competing diagnostic tests will be ranked by a superiority index.

**Results:**

This study is ongoing, and will be submitted to a peer-reviewed journal for publication.

**Conclusion:**

This study will provide a comprehensive evidence summary of CT, MRI, and F-18 FDG PET in detecting the malignant potential of GIST.

## 1 Introduction

Gastrointestinal stromal tumors (GIST) are rare tumors compared with other digestive cancers arising in the muscle layer of the gastrointestinal (GI) tract.^[1]^ Recent studies reported that GISTs present high malignant potential and show a wide spectrum of clinic courses.^[2,3]^ Hence, it is necessary to observe malignant GISTs carefully. Unfortunately, due to the lack of early detection for potential of malignant GISTs, it is hard to make accurate prognosis in patients with malignant GISTs.^[4]^

There have been chemotherapies for the patients with GISTs but part of them are resistant to the treatment, so it is also mandatory to predict the response to treatment for the malignant GISTs. However, preoperative evaluation of malignant GISTs remains difficult for most GISTs are in the submucosa which is hard to assess the stage of malignant GISTs.^[5]^

As we all know, CT, MRI, and F-18 fluorodeoxyglucose (FDG) positron emission tomography (PET) or positron emission tomography or computed tomography (PET/CT) were proved to useful for diagnosing tumor staging in different cancers.^[6]^ CT is a common imaging modality for lots of tumors, besides, for the patients with GISTs, it is also functional to predict the response of treatment.^[7]^ Likewise, MRI is considered efficient to detect malignant GISTs at early stage, which will be helpful for the patients with GISTs receiving chemotherapy treatment.^[8]^ Moreover, F-18 FDG PET or PET/CT has been proved to present high diagnostic accuracy for the patients with malignant potential.^[9,10]^ A recent meta-analysis reported that F-18 FDG PET or PET/CT showed good sensitivity and specificity in predicting malignant potential of GIST.^[11]^ In the recent guideline^[12]^ for the management of GIST, there was no recommended early detection for the malignant potential of GIST, so our study aims to compare the diagnostic accuracy of CT, MRI, and F-18 FDG PET or PET/CT in order to provide better suggestions for the clinic and patients with GIST though network meta-analysis method and to rank these tests using superiority index.

## 2 Methods

### 2.1 Eligibility criteria

#### 2.1.1 Type of study

Eligible studies will meet the following criteria: index tests include either CT, MRI, and F-18 FDG PET or PET/CT or combinations; case–control, cross-sectional, or cohort designs.

#### 2.1.2 Patients

We will include studies which contain patients performed on CT or MRI or F-18 FDG PET or PET/CT to predict malignant potential of GIST. We will exclude studies that provide no sufficient data of diagnostic accuracy. We will put no limitations in age, gender, and nations.

#### 2.1.3 Index tests

We will regard CT, MRI, and F-18 FDG PET or PET/CT as index tests because these tests are usually used to predict malignant potential of GISTs. Study inclusion based on the diagnostic criteria that were used will not be limited while study inclusion based on the quality of CT, MRI, and F-18 FDG PET or PET/CT will be limited.

#### 2.1.4 Reference standards

Definitive histopathology following surgery will be considered as primary reference standard and the clinical follow-up after treatment will be the complementary reference standard.

#### 2.1.5 Outcomes

The primary outcomes are sensitivity, specificity, and diagnostic odds ratios (DOR).The second outcomes are relative sensitivity, relative specificity, and relative diagnostic odds ratio.

### 2.2 Information sources

PubMed, EMBASE.com, CNKI, and CBM databases will be searched until March 2018. The references of relevant systematic reviews/meta-analyses will be searched to identify additional potential studies.

### 2.3 Search strategy

Search strategy of PubMed was as follows:

#1 ((((((“GIST”[MeSH Terms]) OR “gist”[Title/Abstract]) OR “Gastrointestinal stromal tumor”[Title/Abstract]) OR “gastrointestinal stromal tumor” [Title/Abstract]) OR “Gastrointestinal stromal tumors ” [Title/Abstract]) OR “gastrointestinal stromal tumors” [Title/Abstract])

#2 (((((CT [MeSH Terms]) OR ct [Title/Abstract]) OR computer tomography [Title/Abstract]) OR Computed Tomography [Title/Abstract]) OR Computer tomography [Title/Abstract])))

#3 (((((“MRI”[MeSH Terms]) OR“Magnetic Resonance Imaging”[Title/Abstract]) OR “Nuclear Magnetic Resonance Imaging” [Title/Abstract]) OR “nuclear magnetic resonance imaging” [Title/Abstract]) OR “Nuclear Magnetic Resonance Imaging ”[Title/Abstract])

#4 (((((PET/CT [MeSH Terms]) OR Positron Emission Computed Tomography [Title/Abstract]) OR PET [Title/Abstract]) OR F-18 FDG [Title/Abstract]) OR 18F-FDG PET-CT [Title/Abstract])))

#5 #2 OR #3

#6 #2 OR #4

#7 #5 OR #6

#8 #1 AND #7

### 2.4 Study selection and data extraction

We will collect data of interest, which including eligible studies characteristics (e.g., name of the first author, year of publication, country in which the study was conducted, gold standard, and index tests), patients characteristics (male, mean age, sample, method, cutoff level, and risk factors of GIST), and outcomes (SEN, SPE, TP, FP, FN,TN).

Study selection and data extraction will be performed by one reviewer (WKY), and will be checked by other reviewers (GL and PB). Conflict will be resolved by discussion.

### 2.5 Risk of bias

The risk of bias will be independently evaluated by 2 reviewers (WKY and GL) for each study as low, moderate, or high using criteria adapted from Quality Assessment of Diagnostic Accuracy Studies 2 (QUADAS-2).^[13]^ Conflict will be resolved by discussion.

### 2.6 Network meta-analysis

#### 2.6.1 Pairwise meta-analyses

We will perform pairwise meta-analyses for each index test separately. Pooled sensitivity (SEN), specificity (SPE), positive likelihood ratio (PLR), negative likelihood ratio (NLR), diagnostic odds ratio (DOR), and area under the summary receiver operating characteristic curve (AUSROC) will be calculated using bivariate mixed-effects regression modeling with STATA version 12.0 (Stata, College Station, TX). The between-study variance will be calculated var logitSEN and logitSPE.^[15–17]^ The proportion of heterogeneity according to the threshold effect among the included studies will be calculated by the squared correlation coefficient estimated from the between-study covariance variable in the bivariate model.^[18]^ The heterogeneity between each study will be estimated using the *Q* value and the inconsistency index (*I*^2^) test, and the values of 25%, 50%, and 75% for the *I*^2^ will be indicative of low, moderate, and high statistical heterogeneity, respectively.^[19]^

Subgroup analyses for each will be conducted on the basis of the country in which the study was conducted, cutoff level, and risk of bias.

Deek's funnel plot will be carried out to evaluate the potential publication bias when there are more than 10 studies available for an index test.^[20]^

#### 2.6.2 Indirect comparisons between competing diagnostic tests

We will calculate relative diagnostic outcomes between CT, MRI, and F-18 FDG PET by ANOVA model in R software version 3.4.1,^[14]^ including relative sensitivity (RSEN), relative specificity (RSPE), and relative diagnostic odds ratio (RDOR).

#### 2.6.3 Ranking of competing diagnostic tests

Some researchers regard DOR as an indicator of ranking of competing diagnostic tests^[21]^ while the measure might not distinguish between tests with high sensitivity but low specificity or vice-versa. Besides, the superiority index introduced by Deutsch et al^[22]^ provides more weight to tests performing relatively well on both diagnostic accuracy measures and less weight on tests performing poorly on both diagnostic measures or tests performing better on one measure but poorly on the other.^[14]^ The superiority index ranges from 0 to ∞, and tends toward ∞ and 0 as the number of tests to which the target test is superior and inferior increases, respectively, and superiority index tending to 1 the more the tests are equal.^[14]^

## 3 Discussion

CT has been considered as a common diagnostic tool for radiology evaluation in patients with malignant GIST.^[23]^ MRI is also functional and efficient in diagnosing malignant GIST^[24]^ and F-18 FDG PET has unique technique to predict the response to chemotherapy after treatment.^[25]^ Among imaging studies, CT is important for detection and localization, moreover, it plays a vital role in the assessment of extension and follow-up of these tumors in patients with GIST.^[26]^ Recent study reported that MRI plays an important role in detecting differences between imatinib-sensitive and imatinib-resistant GIST tumors,^[27]^ which can improve the treatment in patients with malignant GIST. What is more, F-18 FDG PET is valuable in early evaluation of treatment response and in early detection of malignant potential in patients with GIST.^[28]^ However, it is still unclear which detection will be favorable among the 3 detections for the patients with GIST; hence, we will perform this network meta-analysis to generate evidence and provide suggestions for clinic practice.

## Author contributions

WKY, GL, and PB plan and design the research; WKY, GL, and LCC tested the feasibility of the study; WKY, YH, and CN provided methodological advice, polished and revised the manuscript; WKY and CN wrote the manuscript; all authors approved the final version of the manuscript.

**Data curation:** Kongyuan Wei, Bei Pan, Cuncun Lu, Long Ge.

**Methodology:** Kongyuan Wei, Huan Yang, Cuncun Lu, Long Ge.

**Software:** Kongyuan Wei, Huan Yang, Long Ge.

**Writing – original draft:** Kongyuan Wei, Nong Cao.

**Writing – review & editing:** Kongyuan Wei, Nong Cao.

**Resources:** Bei Pan.

## References

[1] Hassanzadeh-Rad A Yousefifard M Katal S The value of 18F-fluorodeoxyglucose positron emission tomography for prediction of treatment response in gastrointestinal stromal tumors: a systematic review and metaanalysis. J Gastroenterol Hepatol 2016;31:929–35. 26642423 10.1111/jgh.13247

[2] Miettinen M Majidi M Lasota J Pathology and diagnostic criteria of gastrointestinal stromal tumors(GISTs): a review. Eur J Cancer 2002;38 (suppl 5):S39–51. 10.1016/s0959-8049(02)80602-5 12528772

[3] Rosai J Rosai J Gastrointestinal tract. Ackerman's Surgical Pathology. St Louis: Mosby; 1996 645–932.

[4] Joensuu H Vehtari A Riihimäki J Risk of recurrence of gastrointestinal stromal tumour after surgery: an analysis of pooled population-based cohorts. Lancet Oncol 2012;13:265–74. 22153892 10.1016/S1470-2045(11)70299-6

[5] DeMatteo RP Heinrich MC EL-Rifai WM Clinical management of gastrointestinal stromal tumors: before and after STI-571. Hum Pathol 2002;33:466–77. 12094371 10.1053/hupa.2002.124122

[6] Blodgett TM Meltzer CC Townsend DW PET/CT: form and function. Radiology 2007;242:360–85. 17255408 10.1148/radiol.2422051113

[7] Blay JY Bonvalot S Casali P Consensus meeting for the management of gastrointestinal stromal tumors. Report of the GIST Consensus Conference of 20–21 March 2004, under the auspices of ESMO. Ann Oncol 2005;16:566–78. 15781488 10.1093/annonc/mdi127

[8] Gold JS Dematteo RP Combined surgical and molecular therapy: the gastrointestinal stromal tumor model. Ann Surg 2006;244:176–84. 16858179 10.1097/01.sla.0000218080.94145.cf PMC1602162

[9] Van den Abbeele AD The lesions of GIST-PET and PET/CT: a new paradigm for imaging. Oncologist 2008;13 (suppl 2):8–13. 18434632 10.1634/theoncologist.13-S2-8

[10] Basu S Mohandas KM Peshwe H FDG-PET and PET/CT in the clinical management of gastrointestinal stromal tumor. Nucl Med Common 2008;29:1026–39. 10.1097/MNM.0b013e328313bbe7 18987522

[11] Kim SJ Lee SW Performance of F-18 FDG PET/CT for predicting malignant potential of gastrointestinal stromal tumors: a systematic review and meta-analysis. J Gastroenterol Hepatol 2018;33:576–82. 28994187 10.1111/jgh.14015

[12] Judson I Bulusu R Seddon B UK clinical practice guidelines for the management of gastrointestinal stromal tumours (GIST). Clin Sarcoma Res 2017;7:6. 28465823 10.1186/s13569-017-0072-8 PMC5408425

[13] Whiting PF Rutjes AW Westwood ME QUADAS-2 Group. QUADAS-2: a revised tool for the quality assessment of diagnostic accuracy studies. Ann Intern Med 2011;155:529–36. 22007046 10.7326/0003-4819-155-8-201110180-00009

[14] Nyaga VN Aerts M Arbyn M ANOVA model for network meta-analysis of diagnostic test accuracy data. Stat Methods Med Res 2016;pii: 0962280216669182. [Epub ahead of print]. 10.1177/0962280216669182 27655805

[15] Ge L Pan B Song F Comparing the diagnostic accuracy of five common tumour biomarkers and CA19-9 for pancreatic cancer: a protocol for a network meta-analysis of diagnostic test accuracy. BMJ Open 2017;7:e018175. 10.1136/bmjopen-2017-018175 PMC5770961 29282264

[16] Reitsma JB Glas AS Rutjes AW Bivariate analysis of sensitivity and specificity produces informative summary measures in diagnostic reviews. J Clin Epidemiol 2005;58:982–90. 16168343 10.1016/j.jclinepi.2005.02.022

[17] Higgins JP Thompson SG Deeks JJ Measuring inconsistency in meta-analyses. BMJ 2003;327:557–60. 12958120 10.1136/bmj.327.7414.557 PMC192859

[18] Harbord RM Deeks JJ Egger M A unification of models for meta-analysis of diagnostic accuracy studies. Biostatistics 2007;8:239–51. 16698768 10.1093/biostatistics/kxl004

[19] Dwamena BA. MIDAS: statamodule for meta-analytical integration of diagnostic accuracy studies. 2007. Available at: http://econpapers.repec.org/software/bocbocode/s456880.htm Accessed May 8, 2017.

[20] Deek's JJ Macaskill P Irwig L The performance of tests of publication bias and other sample size effects in systematic reviews of diagnostic test accuracy was assessed. J Clin Epidemiol 2005;58:882–93. 16085191 10.1016/j.jclinepi.2005.01.016

[21] Glas AS Lijmer JG Prins MH The diagnostic odds ratio: a single indicator of test performance. J Clin Epidemiol 2003;56:1129–35. 14615004 10.1016/s0895-4356(03)00177-x

[22] Deutsch R Mindt MR Xu R Quantifying relative superiority among many binary-valued diagnostic tests in the presence of a gold standard. J Data Sci 2009;7:161–77.

[23] Choi H Charnsangavej C de Castro Faria S CT evaluation of the response of gastrointestinal stromal tumors after imatinib mesylate treatment: a quantitative analysis correlated with FDG PET findings. AJR Am J Roentgenol 2004;183:1619–28. 15547201 10.2214/ajr.183.6.01831619

[24] Longo DL Dastru W Consolino L Cluster analysis of quantitative parametric maps from DCE-MRI: application in evaluating heterogeneity of tumor response to antiangiogenic treatment. Magn Reson Imaging 2015;33:725–36. 25839393 10.1016/j.mri.2015.03.005

[25] Miyake KK Nakamoto Y Mikami Y The predictive value of preoperative 18F-fluorodeoxyglucose PET for postoperative recurrence in patients with localized primary gastrointestinal stromal tumour. Eur Radiol 2016;26:4664–74. 26852217 10.1007/s00330-016-4242-5

[26] Lupescu IG Grasu M Boros M Gastrointestinal stromal tumors: retrospective analysis of the computer-tomographic aspects. J Gastrointestin Liver Dis June 2007;16:147–51. 17592560

[27] Consolino L Longo DL Sciortino M Assessing tumor vascularization as a potential biomarker of imatinib resistance in gastrointestinal stromal tumors by dynamic contrast-enhanced magnetic resonance imaging. Gastric Cancer 2017;20:629–39. 27995483 10.1007/s10120-016-0672-7 PMC5486478

[28] Malle P Sorschag M Gallowitsch HJ FDG PET and FDG PET/CT in patients with gastrointestinal stromal tumours. Wien Med Wochenschr 2012;162:423–9. 22890522 10.1007/s10354-012-0131-y

